# Recent Progress of In Vitro 3D Culture of Male Germ Stem Cells

**DOI:** 10.3390/jfb14110543

**Published:** 2023-11-08

**Authors:** Jiang Wu, Kai Kang, Siqi Liu, Yaodan Ma, Meng Yu, Xin Zhao

**Affiliations:** 1 Coastal Agricultural College, Guangdong Ocean University, Zhanjiang 524000, China; wuj@gdou.edu.cn (J.W.);; 2 State Key Laboratory for Mechanical Behavior of Materials, Xi’an Jiaotong University, Xi’an 710049, China

**Keywords:** male germline stem cells, *in vitro* culture, niche microenvironment, 3D culture system, biomaterial

## Abstract

Male germline stem cells (mGSCs), also known as spermatogonial stem cells (SSCs), are the fundamental seed cells of male animal reproductive physiology. However, environmental influences, drugs, and harmful substances often pose challenges to SSCs, such as population reduction and quality decline. With advancements in bioengineering technology and biomaterial technology, an increasing number of novel cell culture methods and techniques have been employed for studying the proliferation and differentiation of SSCs *in vitro*. This paper provides a review on recent progress in 3D culture techniques for SSCs *in vitro*; we summarize the microenvironment of SSCs and spermatocyte development, with a focus on scaffold-based culture methods and 3D printing cell culture techniques for SSCs. Additionally, decellularized testicular matrix (DTM) and other biological substrates are utilized through various combinations and approaches to construct an *in vitro* culture microenvironment suitable for SSC growth. Finally, we present some perspectives on current research trends and potential opportunities within three areas: the 3D printing niche environment, alternative options to DTM utilization, and advancement of the *in vitro* SSC culture technology system.

## 1. Introduction

**Figure 1:**
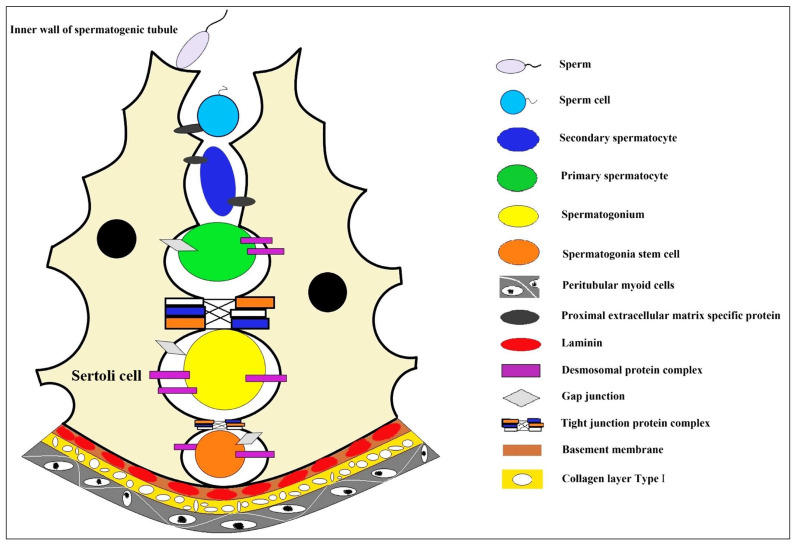
The niche environment during spermatocyte development. In the convoluted seminiferous ducts of the testicles, there are only Sertoli cells and spermatogonium cells (spermatogonium cells are divided into type A_1_ spermatogonium cells, type A_2_ spermatogonium cells, type A_3_ spermatogonium cells, type A_4_ spermatogonium cells, and type B spermatogonium cells according to the sequence they formed after division). A series of cell biological changes in spermatogonium cells are carried out in the niche microenvironment where the Sertoli cells formed. Sertoli cell, a type of cell located in the seminiferous tubules that supports and nourishes the developing sperm cells; sperm, the haploid male gamete after completion of cell morphological deformation; sperm cell, the haploid male gamete with incomplete cell morphology; secondary spermatocyte, small in size and located on the inner side of the primary spermatocyte, closer to the lumen of the seminiferous tubule; primary spermatocyte, one type of spermatocytes, primary and secondary spermatocytes are formed through the process of spermatocytogenesis; spermatogonium, an immature male germ cell that divides to form many spermatocytes; spermatogonia stem cell, a type of adult stem cell located on the basal membrane of the tubule that is capable of self-renewal to maintain a constant number of its own population and directional differentiation to produce spermatocytes; peritubular myoid cells, the main cell components of the basement membrane of the seminiferous tubule create a unique microenvironment for germ cell development; proximal extracellular matrix specific protein, highly specialized extracellular matrix proteins, including type IV collagen, laminin, nestin, heparan sulfate proteoglycan, etc.; desmosomal protein complex, a cell adhesion complex that connects adjacent epithelial cells to each other and attaches keratin filaments to the surface of the epithelial cells; gap junction, a way of cell connection that allows the free passage of small molecules with molecular weights less than 1.5kD to maintain the stability of the internal environment and the normal physiological function of the tissue cells; tight junction protein complex, mainly exists between epithelial cells and endothelial cells, making adjacent cell membranes close together to form a physical barrier structure around the cell; basement membrane, a thin connective tissue membrane that separates the epithelial cell layer from the underlying stromal layer; laminin, a major protein in the basal lamina (one of the layers of the basement membrane), a protein network foundation for most cells and organs; collagen layer type l, the main component of the basement membrane of parenchymatous organs also plays a major role in the formation of specific extracellular microenvironments.

The production of mature male gametes depends on the continuous self-renewal and differentiation of male germline stem cells (mGSCs), also named spermatogonial stem cells, SSCs) [1]. The development of an efficient and convenient artificial culture system makes it possible for the cryopreservation and genetic modification of SSCs, which also lays the foundation for artificial spermatogenesis and sperm production and has enormous potential practical value; for example, SSCs from boys undergoing cancer treatment may be cryopreserved to protect their germ cells [1,2]. In the spermatogenic epithelium, the only somatic cells that spermatogenic cells contact are testicular Sertoli cells [3,4]. The unique internal environment formed in the spermatogenic epithelium could maintain spermatogenesis and provide a unique niche environment for the genesis and differentiation of germ cells [3,5]. In this microenvironment, germline stem cells, spermatogonium, primary spermatocyte, spermatocyte, and sperm are formed one after another (Figure 1). Therefore, artificial technology to simulate such an *in vitro* microenvironment is the primary condition for the 3D culture of SSCs. On this basis, it will be of great value to develop a new *in vitro* 3D cell culture system to induce the differentiation of SSCs into functional male germ cells [6,7].

Sertoli cells are the only somatic cells in contact during the proliferation and differentiation of SSCs. Therefore, in the early SSC culture system, pre-cultured primary Sertoli cells were used to build the *in vitro* microenvironment of SSCs. Later, primary mouse embryonic fibroblast cells (MEF cells) and STO cells (a kind of fibroblast isolated from the embryo of a mouse) will be used [8,9,10,11]. The primary cells have the problems of unstable cell character and inconvenient acquisition, and the cell line has some differences that exist compared to the primary cell. These problems have prompted researchers to develop more convenient and efficient materials for the microenvironment construction of *in vitro* SSC culture. The microenvironment formed by the extracellular matrix plays some critical roles in regulating cellular morphology, proliferation, and differentiation, and the extracellular matrix derived from natural healthy tissues could even support the growth of organoids that occur *in vivo* under *in vitro* culture conditions [12]. Therefore, researchers used physical, chemical, and biological technologies to advance a variety of natural substrates (such as collagen, polysaccharides, and glycosaminoglycan) to construct a microenvironment for the growth of SSCs *in vitro*, to achieve long-term culture and technical development and utilization of SSCs *in vitro*, such as induction and differentiation into functional sperm cells *in vitro*, preparations of male germ seed cells that can be used for transgene animals, etc.

This review introduces the growth niche environment of mGSCs and the research progress of *in vitro* 3D culture technology of SSCs simulating this microenvironment. First, the microenvironment of SSCs and spermatocyte development are summarized (Figure 1). Then, the review focuses on the scaffold-based culture and 3D printing cell culture techniques for SSCs, decellularized testicular matrix (DTM), and other biological substrates used in different combinations and methods to construct the *in vitro* culture microenvironment of SSCs. Finally, we summarize our views on recent progress and potential opportunities in three areas: 3D printing niche microenvironment, new alternatives to DTM, and the *in vitro* SSC culture technology system.

## 2. The Niche of Spermatogonial Stem Cells (SSCs)

SSCs are the basis and initial cells of spermatogenesis and the only adult stem cells that transmit genetic and epigenetic information in adult males [13]. SSCs proliferate and differentiate in a “niche” structural space formed by Sertoli cells [14]. Therefore, when cultivating SSCs *in vitro*, the research will encounter two problems: 1. Sertoli cells are sufficiently stable to provide the niche environment for the long-term *in vitro* culture of SSCs continuously. 2. SSCs are difficult to spontaneously immortalize, while transgenic-modified SSCs lose part of their stem cell characters, making them difficult to use from bench to bed.

### 2.1. The Spermatogonial Stem Cells

Spermatogonial stem cells (SSCs) are the adult stem cells on the basement membrane of the seminiferous tubule, and they can self–renew and differentiate eventually into sperms. SSCs are considered an ideal model for studying spermatogenesis, meiosis regulation, and cell reprogramming *in vitro* [15]. However, with the increase in individual age, especially in the *in vitro* culture, SSCs are engulfed in cell senescence problems such as decreased proliferation ability and loss of stem cell characteristics, which limit their experimental and clinical utilization [16].

### 2.2. The Microenvironment of Spermatocyte Development

With the unceasing research on stem cells, the effects of niche environments on stem cells in living human tissue are of increasing concern [17]. Stem cells have enormous research value due to their pluripotency. Still, it is challenging to maintain their pluripotency *in vitro* or induce their directed differentiation, so it is necessary to study the composition of the cellular niche environment and its regulatory mechanism on stem cells [18]. Niches influence the fate of stem cells through direct or indirect interactions with the cells through several factors, such as the extracellular matrix, cytokines, growth factors, pH, and ions among the stem cells and neighboring cells [19]. For example, hematopoietic stem cells (HSCs) maintaining quiescence are supported by the specialized “niche” microenvironment in the bone marrow [18] and, hair follicle stem cell (HFSC) activity is modulated in a dual-component niche formed by arrector pili muscles and sympathetic nerves [19]. Cell niches are often in a state of dynamic microenvironment to maintain the vitality, homeostasis, and repair capacity of cells and tissues throughout the life cycle of the organism [20]. However, specific regulatory mechanisms need extensive research.

After the discovery of the “stem cell niche” in the hematopoietic system, similar structures have been found in other tissue systems, and the niche also exists in the testicular tissue (Figure 1). The SSC niche is a semi-open isolation system in the testis, with specific quantitative regulation and age-dependent characteristics. Some key signaling pathways are involved in the spermatocyte development niche, such as the Plzf regulatory network, GDNF-GFRa1 pathway, and others [21,22,23]. Two endogenous factors, Nanos2 and Plzf, regulate the self-renewal of SSCs [24]. Sertoli cells in the SSC niche also secrete glial cell-derived neurotrophic factor (GDNF) to handle spermatogonial renewal [25]. Sertoli cell defection often leads to infertility [26]; for example, mice with Sertoli cell defects showed increased male fertility and lower sperm counts [27]. Sertoli cells have the most critical roles in regulating SSCs to proliferate or differentiate the microenvironment in seminiferous tubules. The composition of this microenvironment is related to the intercellular junction complex in close contact with SSCs. Meiotic and postmeiotic germ cells after SSC differentiation are isolated by Sertoli–Sertoli junction complexes in the lumen of the seminiferous tubule, respectively [28].

## 3. The 3D Cell Culture of SSCs

Three-dimensional cell culture could supply an artificial space mimicking *in vivo* conditions, which enables cells to migrate and grow in a three-dimensional environment [29]. Three-dimensional culture technology also could preserve the physical and structural basis of the cell microenvironment *in vivo* and demonstrate the advantages of intuitiveness and conditional control of cell culture [30,31]. These culture models are constructed to intercellular and cell–extracellular matrix interactions, to mimic the physical, nutritional, and metabolic environment of the microenvironment in which cells reside in the body [30,31,32,33].

### 3.1. Scaffold-Based 3D Cell Culture for SSCs

#### 3.1.1. Scaffold-Based 3D Cell Culture

*In vitro* 3D cell culture is used in various cell biology studies, such as different stem cells, cancer cells and somatic cells. It is becoming increasingly popular with researchers [34]. Scaffold-based 3D culture technology could provide good physical support for cells, whether simple mechanical structures or analogs like extracellular matrix, on which cultured cells could achieve aggregation, proliferation and migration activities [35,36]. In the scaffold-based 3D cell culture, cells are grown in a matrix with specific physicochemical properties (Such as the matrix hydrophilicity, hydrophobicity, ultrastructure, spatial conformation, biological activity and other properties), which affect the properties of the cells. The scaffold could be natural or synthetic and applied for exerting important functions based on adhesion, hardness and load capacity. In experimental studies, some growth factors (such as GDNF, bFGF and others) or bioactive molecules (such as D-serine, retinoic acid and others) could also be embedded in the cell culture scaffold, which could enhance SSCs’ proliferation or promote cell differentiation [2,26,37,38]. Therefore, the selection of scaffolds for *in vitro* 3D culture of SSCs should take into account many factors, such as the material properties and the pore size, rigidity, flexibility and stability of the cultured matrix materials, especially the biological characteristics such as cell compatibility and adhesion [39,40].

**Figure 2:**
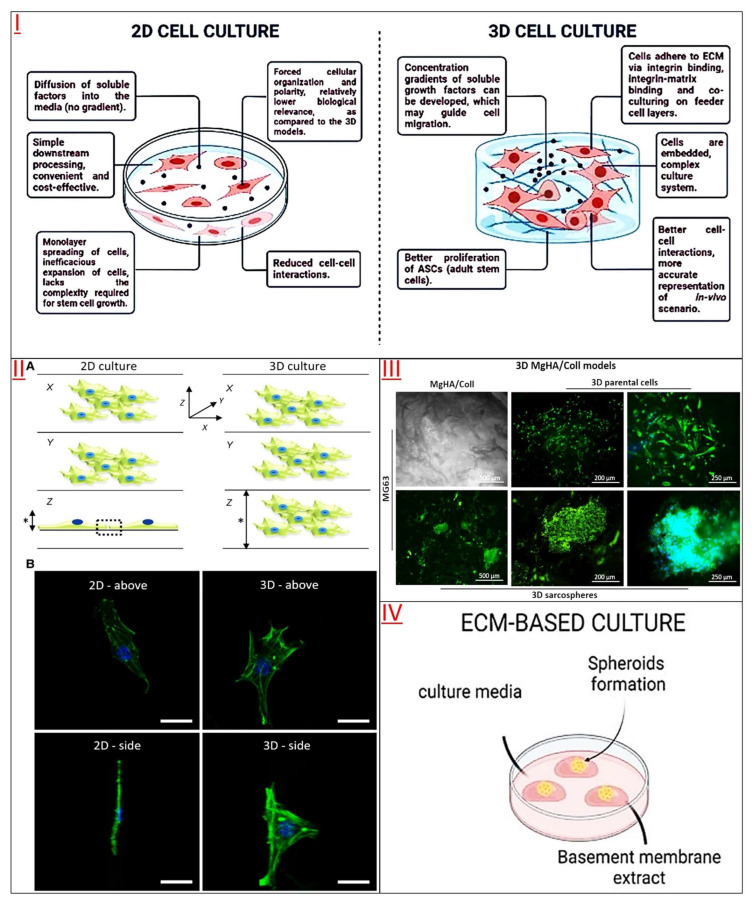
Three-dimensional cell culture facilitates cell proliferation, differentiation, and functional maintenance by providing a suitable niche environment. (**I**) Schematic of the main differences between 2D and 3D cell culture models. In the 3D model, soluble cell growth factors could form concentration gradients, and the cells also show better intercellular interactions. Reprinted from Ref. [54]. (**II**) Three-dimensional culture mode is beneficial to maintaining normal cell morphology and structure. (**A**) Visualization of 3D (X, Y, Z) cells. In the 3D model, the cells maintain a more natural stereoscopic structure and normal size (right), and the range of intercellular contact is omni-stereoscopic. Additionally, compared to conventional 2D monolayer cultures, the total height (*) of 3D cultures is more versatile, depending on the utilized 3D cell technology, and can be engineered to create multi-layered tissue-like structures. In a 2D environment, cell interactions are limited to the periphery of a single plane (left, dotted box), whereas in the 3D model, intercellular contact spans throughout. (**B**) Confocal images of individual fibroblasts between 2D and 3D cell culture models. Phalloidin staining shows the main structure of the F-actin cytoskeleton, 4′,6-diamino-2-phenylindole (DAPI) staining shows the nucleus, and the cells have a more normal cell structure in the 3D culture model (right), while the cell morphology appears flat in the traditional 2D culture (left). Scale = 10 μm. Reprinted from Ref. [55]. (**III**) Fluorescence photograph of MG63 cells in a 3D MgHA/Coll model. Actin filament fluorescence analysis also confirmed a good interaction between cells and ECM in the 3D model, with the nucleus in blue (DAPI) and the F-actin filament in green (Phalloidin). Reprinted from Ref. [56]. (**IV**) Three-dimensional culture methods of organoids based on extracellular matrix, which embed cells in basement membrane extract (BME) to promote the formation of organoid structures by mimicking the microenvironment in which cells live in the body. Adapted from Ref. [57].

Hydrogels comprise cross-linked poly chains or complex networks of natural or synthetic protein molecules [41]. Naturally derived hydrogels carry a large amount of water, which makes them have very similar biological and physical properties to natural tissue structures, so they are widely used as efficient 3D cell culture substrates for cell culture *in vitro* [12]. Hydrogels could be used directly in cell culture alone or in combination with other biomaterials (such as biological scaffolds, matrix membranes, or microfluidics) to adapt to the needs of specific cells. Hydrogels also used to coat the cell culture support or enclose/clamp cells in the matrix [42,43,44]. Naturally derived hydrogels are composed of bioactive factor or extracellular matrix (ECM) components, such as chito-oligosaccharides, collagen, laminin in cell culture [45,46,47,48,49]. These natural endogenous active factors embedded in hydrogels could maintain the survival, proliferation, differentiation and biological function of different cells [50,51,52,53], and these gels themselves are biocompatible and bioactive, which is conducive to the completion of cell functions (Figure 2 and Table 1, Table 2 and Table 3).

#### 3.1.2. DTM-Based Scaffold Culture for SSCs

With the development of cell and tissue engineering, biological scaffolds have been applied in many cases due to their excellent biocompatibility, bioactivity and mechanical properties [58]. Decellularized extracellular matrix (dECM) scaffold is a biological scaffold formed from organism tissue/organ by removing cells and other immunogenic components through acellular technology. The dECM scaffolds mainly comprise extracellular macromolecules, such as collagen, fibronectin and laminin. In addition, tissues and organs could retain their original physical and chemical signals and biological characteristics after decellularization, providing an excellent physical support matrix for the subsequent 3D culture of cells *in vitro* [59]. dECM scaffold also has been widely used in tissue and organ repair *in vivo*. Compared with synthetic materials, dECM could retain the microenvironment and natural 3D structure of the original tissue, and play an essential role in migration, adhesion, differentiation and proliferation for transplanted cells, due to its good biological activity, biocompatibility and degradability [58,59,60,61,62,63,64,65]. The 3D structure of the dECM scaffold could create an *in vivo*-like niche to form an *in vitro* testicular co-culture model with specified cell density and ECM composition for spermatogonial cells [66].

The DTM is an appropriate and effective layer for the proliferation and differentiation of SSCs (Table 1). The DTM supplemented with D-serine and glutamic acid has been shown to provide a suitable microenvironment for the survival of SSCs [67]. In addition, spermatogonium cells on DTM hydrogel scaffolds were more easily differentiated by N-methyl-D-aspartate (NMDA) receptor agonists [37]. Some scholars have also studied the differentiation and apoptosis of germ cells in DTM culture and established an *in vitro* culture model that could induce mature sperm [68]. Furthermore, after long-term culture of SSCs on the DTM layer prepared by sheep testis, the expression of meiosis-related genes in the cells was significantly up-regulated [69]. Therefore, DTM could not only be used to explore the optimal culture conditions of spermatogonial stem cells but also to induce differentiation of SSCs, which could increase the expression of premeiotic, meiotic and postmeiotic genes [70]. Although there is a lack of testicle-specific topography when cultured *in vitro*, DTM does provide an essential niche environment for cultured SSCs to maintain their cellular characteristics [71]. Moreover, due to the preservation of crucial extracellular matrix components such as collagen, fibronectin and laminin in DTM, makes DTM a promising biological material for the development of *in vitro* culture and induction of spermatogenesis of spermatogonial stem cells, treatment of various types of male fertility disorders, or for the development of new animal reproduction techniques [72].

**Table 1:** Summary of types of male reproductive stem cells cultured in DTM-3D *in vitro*.

Culture Material	Cell Type	Species	Main Biological Findings	Reference
Sertoli cells and Leydig cells with extracellular matrix (ECM) composition	SSCs	Mice	The coculture 3D structure prepares an *in vivo*-like niche and supports the proliferation of germ cells.	[66]
An artificial testicular tissue using a DTM -hyaluronic gel matrix	SSCs	Mice	The decellularized testicular matrix supplemented with D-serine and glutamic acid could provide an appropriate niche environment for the proliferation of SSCs.	[67]
DTM hydrogel	SSCs	Mice	The differentiation of spermatogonia could be regulated by D-serine in the 3D culture system.	[37]
Azoospermia tissue DTM	SSCs	NMRI mice	The presence of D-serine and retinoic acid has a positive effect on spermatogenesis in the 3D culture system.	[68]
Sheep DTM	SSCs	Human	The natural structure of DTM prepares the suitable niche environment for the spermatogenesis *in vitro*.	[69]
Sheep DTM	SSCs	Human	SSCs culture in DTM created a way of demonstrating spermatogenesis *in vitro*.	[70]
Human DTM	SSCs	Human	Despite the lack of testis-specific tissue structure, three-dimensional culture *in vitro* could harbor spermatogonium cells and provide their essential niche environment, so that these spermatogonium cells retain specific functions in long-term culture. These findings also open up the possibility of recreating the testicular microenvironment (such as organoid tissue) from primary testicular cells *in vitro*.	[71,72]
ECM	SSCs	Rats	In the 3D rat testicular cell co-culture model, the proliferation, differentiation, and androgen receptor (AR) protein expression of spermatogonia cells could be regulated by experimental methods.	[73]
Human DTM	SSCs	Human	The niche microenvironment created by the multicellular 3D testis organoid model could maintain the long-term viability of spermatogonia cells. It could also promote the differentiation of SSCs into postmeiotic germ cells, simulating the process of spermatogenesis *in vivo*, so that about 0.2% of SSCs differentiate into sperm cells.	[74]

A 3D co-culture model with testicular cells could be used for testicular toxicity screening, which was used to evaluate the effects of various reproductive toxic chemicals on spermatogonium proliferation and differentiation and even spermatogenesis [73]. Another developed human testicular three-dimensional organoid culture system also showed good evaluation function in cell culture *in vitro*, and its IC50 value was significantly higher than that of the two-dimensional culture system after treatment with four chemotherapy drugs [74].

**Figure 3:**
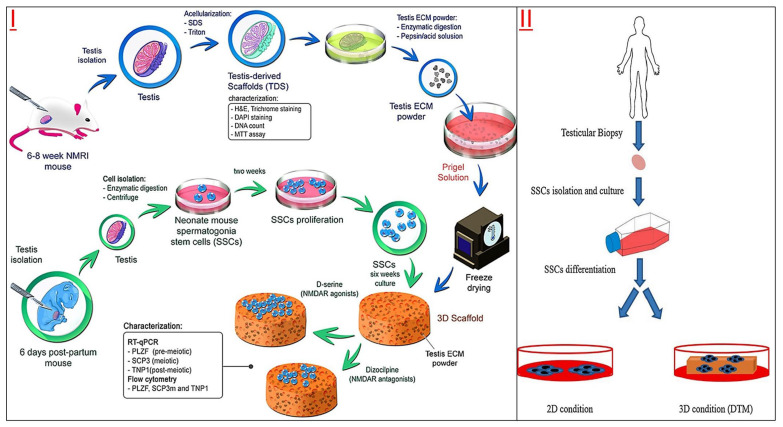
DTM-based scaffolds for SSCs culture. (**I**) SSCs culture on a DTM-based hydrogel scaffold. Reprinted with permission from Ref. [37]. Copyright 2023 Elsevier. (**II**) The DTM-based hydrogel scaffold prepares a niche environment for the spermatogenesis *in vitro*. Reprinted with permission from Ref. [69]. Copyright 2023 Elsevier.

The use and in-depth study of the DTM prompts us to pay attention to the specific components of testicular ECM and elucidates its roles in spermatogenesis, as adequate nutritional and microenvironmental support is essential for SSCs self-renewal and differentiation *in vitro*. DTM has the characteristics of ideal tissue scaffolds: a complex composition, many biomatrix active components, and a unique tissue-specific structure, and these spermatogonial stem cells are fundamental *in vitro* culture. The preparation for DTM should first consider the animal species and physiological state as the testicular source. Generally, healthy adult males are selected as organ donors. Some researchers have tried DTM from xenogeneic animal donors and found that it could maintain SSC self-renewal and spermatogenesis *in vitro*. However, the preparation process for DTM is still relatively complex. Using eluents (including ionic eluent SDS and non-ionic eluent triton X-100), low or high permeability solution, acid-base, organic solvent, etc., to achieve a good decellulatory effect. However, there are also problems of eluent toxicity. It takes a long elution time to remove the residue in tissues and organs and reduce the toxicity caused by the eluent, so it is necessary to develop some convenient and non-toxic *in vitro* culture of biomaterials matrix and culture system (Figure 3).

#### 3.1.3. Non-DTM-Based Scaffold Culture for SSCs

**Figure 4:**
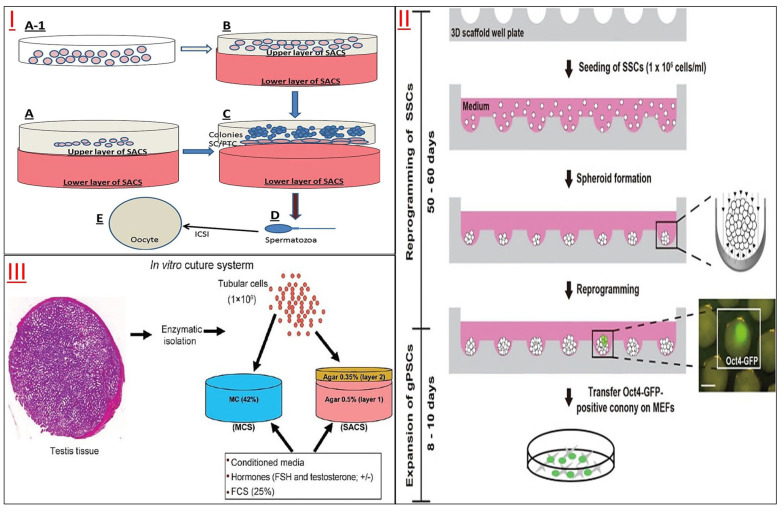
Non-DTM-based scaffold for SSCs culture. (**I**) Spermatogenesis in an Artificial Three-Dimensional. The Sertoli cells were cultured (**A-1**) and placed on SACS (soft agar culture system) or cultured directly on SACS (**A**) to form an artificial 3D environment (**B**). SSCs were placed in a 3D culture system to form clones (**C**) and further differentiate into sperm (**D**), and then sperm-egg union was completed by ICSI technology (**E**). Reprinted with permission from Ref. [98]. Copyright 2023 Oxford University Press. (**II**) Diagram of OCT4^+^ generative pluripotent stem cells generated by SSCs on a 3D scaffold well plate. Reprinted from Ref. [79]. (**III**) Schematic diagram of two 3D culture systems of SSCs *in vitro*, MCS and SACS. MCS consists of methylcellulose, fetal bovine serum and medium. SACS consist of two layers: a solid lower layer (layer 1) 0.5% (*w*/*v*) agar powder consisting of fetal bovine serum and medium, and a soft upper layer (layer 2) 0.35% (*w*/*v*) agar powder. Reprinted with permission from Ref. [84]. Copyright 2023 Wolters Kluwer—Medknow.

With continuous research on the feeder layer, some artificial matrix adhesives have been tried to replace DTM for *in vitro* culture of SSCs (Figure 4 and Table 2). Alginate gel encapsulation culture of SSCs could significantly increase the expression of pluripotent genes Oct4, Sox2 and Nanos2 and promote the aggregation of cell clones [75]. In addition, SSCs could even be converted into induced germline stem cells (iGSCs) in 3D cell culture, reconstructing the ovulation process of SSCs [76]. Another agar/polyvinyl alcohol nanofiber (PVA) scaffold showed a good promotion effect on the proliferation and differentiation of neonatal mouse SSCs. It could improve SSC differentiation into meiosis and post-meiosis cells [77]. The platelet-rich plasma (PRP) scaffold with expression of glial cell line-derived neurotrophic receptor α1 (GFRa1) and c-Kit revealed a significant *in vitro* proliferation of SSCs [78].

2D culture has limitations in intercellular connectivity, cell shape, property maintenance, etc. Studies have confirmed that a 3D culture system with a biological substrate is more conducive to long-term feed-free SSC culture and induction of SSC pluripotency [79]. Most simply, the host testicular fragments were used as a three-dimensional organ culture, and the proliferation and differentiation of SSCs were normal *in vitro* culture [80]. Another three-dimensional soft agar culture system (SACS) could successfully culture mouse SSCs and produce sperm because SACS could simulate the reconstruction of a niche microenvironment capable of regulating cell colony proliferation and differentiation [81], monolayer SSCs or testicular tissue fragment SSCs could also be cultured using agarose constructed 3D conditions [82] to induce spermatogenesis in 3D culture *in vitro* [83]. It was confirmed that SACS and methylcellulose (MCS) formed a particular three-dimensional microenvironment that could simulate the germ cell niche environment in the spermatogenesis of mouse SSCs *in vitro* [84]. A collagen gel culture system combined with somatic testicular cells could also simulate the microenvironment of spermatogenic epithelium and induce spermatogenesis *in vitro* [85]. However, 3D nanofiber scaffolds, which act similarly to ECM/basement membrane, have been shown to enhance the proliferation and self-renewal of SSCs [86]. The proliferation and differentiation of spermatogonium in primates and rodents could be induced by 3D AGAR and MCS culture systems [87]. Even SSCs collected from patients with obstructive azoospermia could continue to proliferate on lamin-coated plates for two months or longer, maintaining their cellular characteristics, proliferation and differentiation [88].

Currently, the 3D culture system of SSCs involving feeder layer cells is still being improved. In SACS, the proliferation of human SSCs could be promoted by co-culture with Sertoli cells, thus preparing a sufficient number of cells for autologous transplantation and *in vitro* spermatogenesis [89]. In other animals, when goat SSCs were cultured *in vitro* culture system, stable SSC clones could be maintained by co-culture with the Sertoli cell feeding layer [90], the STO cells (a fibroblast cell line that was isolated from the mouse embryo) have also been shown to be suitable layers for proliferation of bovine SSCs *in vitro* [91], and attaching Sertoli cells to a culture dish coated with mandala lectin (DSA) also helped establish a long-term culture system for buffalo spermatogonium [92]. Studies on STO feeding layer or gelatin-coated Petri dish confirmed that SSCs-STO co-culture could better maintain the characteristics and cell proliferation of SSCs [93], and the three-dimensional culture of SSCs mixed with alginate gel and Sertoli cells could also effectively promote cell proliferation and maintain SSC stemness [94]. A novel 3D testicular cell co-culture model could make spermatogonial cells present better cell structure *in vitro* and facilitate intercellular communication between different cell types [95]. A microporous culture system of the STO cell feeding layer could encourage the formation of SSC cell clones’ structure more efficiently [96].

**Table 2:** Summary of types of male reproductive stem cells cultured in no-DTM-3D *in vitro*.

Culture Material	Cell Type	Species	Main Biological Findings	Reference
Alginic acid	SSCs	Mice	Alginate scaffold structure could maintain the morphology and cell density of SSCs for a long time, and promote the expression of pluripotent genes.	[75]
Gonadal somatic cells and transwell-COL membranes	SSCs	Mice	In a 3D organoid culture system, SSCs are transformed into induced germline stem cells (iGSCs) with maternal imprinting patterns through transgenic manipulation.	[76]
Agar/polyvinyl alcohol nanofiber scaffold	SSCs	Mice	In the three-dimensional culture system of AGAR/PVA scaffold, the differentiation of mouse SSCs into spermatoblasts could be enhanced synergically with the medium supplemented with growth factors.	[77]
PRP + CaCl_2_	SSCs	Human	PRP scaffold could reconstruct a suitable niche environment for the *in vitro* proliferation of SSCs.	[78]
3D Stemfit^®^ culture dishes (3D scaffold)(MicroFIT, Seongnam, Korea)	SSCs	Mice	Using 3D scaffolds, SSCs could be reprogrammed to become gPSCs without biological substrates.	[79]
Agarose gel stands	SSC-LCs derived from iPSCs	Mice	iPSCs could hom in a three-dimensional testicular niche environment, which plays a crucial role in inducing iPSCs to differentiate into spermatogonial stem cell-like cells.	[80]
Human Sertoli cells	SSCs	Human	3D culture could significantly increase the number and size of SSCs clones and the expression of spermatogonial marker genes.	[81]
Agarose	SSCs	Mice	Agarose 3D culture induced spermatogenesis process *in vitro*.	[82]
Methylcellulose Culture System (MCS)	SSCs	Mice	The MCS 3D culture system could induce the differentiation of normal immature spermatogonium into meiotic and postmeiotic cells and produce sperm-like cells.	[83]
MCS AND SACS	STC	Rhesus monkeys	The 3D culture system could partially simulate the microenvironment in the seminiferous tubules and promote the differentiation of type A spermatogonium to spermatocyte.	[84]
Collagen gel matrix	SSCs	Balb-c mice	The three-dimensional culture system of collagen gel provided a microenvironment that mimics the spermatogenic epithelium and could induce spermatogenic processes in spermatogonium *in vitro*.	[85]
Poly L-lactic acid(PLLA) nanofiber scaffold	SSCs	Mice	PLLA could promote the formation of spermatogonia clones and induce cell differentiation during culture	[86]
Methylcellulose(MCS)	SSCs	Human	3D MCS culture system could induce spermatogenic processes of spermatogonium isolated from living tissue	[87]
Sertoli cells in SACS + laminin + growth factors	SSCs	Human	Laminin could replace Sertoli cells to construct a three-dimensional culture system, that enables specific spermatogonium to self-renew or differentiate	[89]
Sertoli cell feeder layer in goat	SSCs	Sheep	In a culture system with Sertoli cells as feeder layers, SSCs could emerge as cell clones after a short time culture.	[90]
SIM mouse(Sando’s inbred mouse) embryo-derived thioguanine and ouabain resistant (STO), and a laminin-coated plate.	SSCs	Bovine	In the culture system of the STO feeder layer, SSCs could form many cell clones and express SSCs marker genes at a high level. In the three-dimensional culture system of laminin, the pluripotent genes of SSCs were highly expressed.	[91]
DSA lectin-coated dishes with the attachment of Sertoli cells.	SSCs	Buffalo	DSA lectin-coated dishes supported long-term maintenance and self-renewal of SSC-like cells.	[92]
NMRI Mouse STO and Growth Factors	SSCs	Mice	The SSC-STO co-culture provided a microenvironment for efficient maintenance and proliferation of SSCs.	[93]
3D alginate hydrogel with Sertoli cells	SSCs	Mice	Alginate gel three-dimensional culture could promote the proliferation of SSCs and the maintenance of cell stemness and improve the survival rate of SSCs transplantation.	[94]
Sertoli, and Leydig cells	SSCs	Murine C18-4	The testicular co-culture model could make spermatogonial cells present better cell structure *in vitro* and promote intercellular communication between different cell types.	[95]
Membrane-bottomed microwell array added to Transwell insert + STO cells	SSCs	Mice	The microporous culture system of the STO cell feeding layer could promote the formation of SSCs cell clonoid structure more efficiently.	[96]

Now, some new 3D bioprinting technologies have been applied for SSC culture *in vitro*. The 3D bioprinting process could better maintain testicular cell vitality, survive the cell types of reproductive cells and maintain their cell characteristics, and these advantages showed excellent application potential in testicular germ cell culture and induction of spermatogenesis [97].

### 3.2. 3D Printing Cell Culture for SSCs

#### 3.2.1. 3D Printing Cell Culture

Three-dimensional printing is a type of manufacturing process in which materials such as plastic or metal are deposited in layers to create a three-dimensional object from a digital model [99]. Cell bioprinting (3D bioprinting) is an advanced technology derived from 3D printing in recent years for the construction of multicellular systems *in vitro* [100,101]. Three-dimensional bioprinting is an organic combination of rapid prototyping and biological manufacturing, which could solve problems challenging to be solved by traditional tissue engineering [102]. Three-dimensional bioprinting could be divided into two types based on how the supporting material is formed: the scaffold is printed first, and then the cells are implanted, or the cells are mixed with the scaffold and printed together [103,104,105]. In the process of cell printing, cells (or cell aggregates) and sol (precursor of hydrogel) are placed in the nozzle of the printer at the same time, the deposit position of cell droplets is controlled by the computer, then printed point by point in the specified place, layer by layer, to form a 3D multicellular/gel system (Figure 3 and Table 3). Three-dimensional bioprinting has good material compatibility, and various cells with different functions, extracellular matrix, cell growth factors and biodegradable polymers could be printed as support materials. Therefore, some researchers have used 3D bioprinting technology to solve the problem of a significant reduction in the number of spermatogonium after ITT transplantation in frozen preserved immature testicular tissue (ITT) [106]. ITT was embedded in a hydrogel containing VEGF nanoparticles, and immunohistochemical assessment of seminiferous integrity, hematopoietic reconstruction, and spermatogonial recovery showed that alginate gels had significantly higher spermatogonial recovery rates and had the potential to promote cryopreservation in tissue transplantation [107]. Three-dimensional bioprinting technology has made remarkable achievements in recent years, but there are still many challenges in manufacturing reproductive system tissues. For example, the cost of biological 3D bioprinting is generally high, and it is difficult to achieve wide application. The isolation and culture of cells for 3D bioprinting is complex and requires specialized cell culture technicians. For the specific structural requirements of the microenvironment for spermatogonial stem cell proliferation and differentiation, the printing materials also have good biocompatibility, biomechanical properties, etc. [108,109]. Therefore, further research is needed to create more alternatives with 3D bioprinting that could be used for cell engineering production [110]. While it is confirmed that 3D culture could maintain the differentiation ability of the spermatogonium, it is more important to carry out more studies to further reduce senescence and apoptosis of spermatogonia during *in vitro* culture and save more seed cells for subsequent research and development [106].

#### 3.2.2. 3D-Printed Scaffold Culture for SSCs

**Figure 5:**
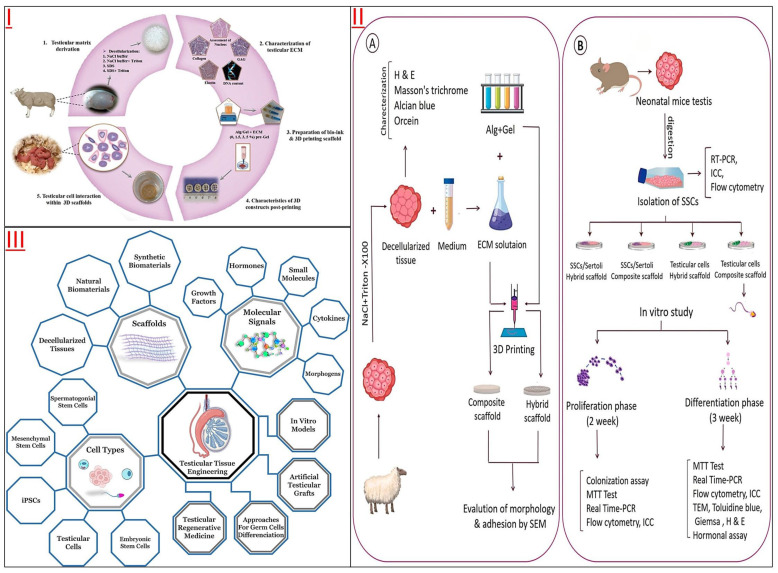
(**I**) Experimental design diagram of DTM for SSCs’ 3D printing culture. Reprinted with permission from Ref. [38]. Copyright 2023 Royal Society of Chemistry. (**II**) Three-dimensional printing scaffold culture of mouse SSCs and *in vitro* production of mouse morphological sperm. (**A**) Three-dimensional printing scaffold fabricated on DTM extracted from ram testis. (**B**) The cell biology experimental detection of mouse SSCs on 3D printing scaffolds. Reprinted with permission from Ref. [111]. Copyright 2023 Elsevier. (**III**) Schematic diagram of the research branches of tissue engineering and cell engineering related to testicular tissue. Among the three important branches (molecular signals, cell types and scaffolds), scaffolds could be divided into three sub-directions: synthetic biomaterials, natural biomaterials, and decellularized tissues. Reprinted from Ref. [113].

Some studies have found that 3D-printed scaffolds could provide a comfortable environment for the survival, proliferation, and even spermatogenesis of SSCs (Figure 5 and Table 3). The DTM of male testicular tissue was prepared as bio-ink printing artificial testicular tissue, and the testicular spermatogonia cultured on the DTM scaffold showed good cell viability, adhesion ability, and up-regulated expression of premeiotic marker genes [111]. Another improved 3D DTM scaffold is a spherical shape with a completely curved seminiferous tubule-like structure called a testicular organoid (TO). These 3D-printed mouse TOs could support the long-term survival of spermatogonia and induce their differentiation to the meiosis phase [112]. Further studies also found that 5% DTM 3D-printed scaffolds could induce spermatogenesis *in vitro* due to their uniform surface morphology, high cell adhesion, and biocompatibility to spermatogonia [38].

**Table 3:** Summary of types of male reproductive stem cells cultured in 3D bioprinting *in vitro*.

Culture Form	Culture Material	Cell Type	Species	Main Biological Findings	Reference
Cells no-printed	Ram ECM + Alg-Gel	SSCs	NMRI mice	Testicular cells cultured on T-ECM-enriched scaffolds showed high cell viability and expressed genes related to spermatogonial differentiation.	[111]
	Agarose gel	SSCs	Mice	In the double-layer scaffold culture system, the organoid structure formed by the mixture of primary testicular cells and germline stem cells showed a curved spermatotubule-like microenvironment structure.	[112]
	Ram DTM with alginate–gelatin	SSCs	Mice	The 3D-printed scaffold of 5% T-ECM could enable good adhesion of SSCs and promote their cell survival *in vitro*.	[38]
Cells printed	PCL powder and gelatin	SSCs	Human	The PCL powder and gelatin nanocomposite scaffold could provide a suitable microenvironment for self-renewal of SSCs	[114]
	Hydrogels comprised of alginate and gelatin	MCS	Sheep	The 3D-printed culture system of 7%Alg-8%Gel mixture could maintain good cell viability, and the cell function could be kept for a long time when incubated with the cell-loaded hydrogel.	[115,116]
	AGC-10 matrix bioink composed of alginate and rat tail-derived collagen	SSCs	Patients with NOA	After bioprinting, human testicular cells could proliferate well, have high cell viability, and maintain their biological function.	[97]

#### 3.2.3. 3D-Printed Scaffold-SSCs Matrix

Three-dimensional bioprinting through mixing cells with matrix glue could improve the cell microenvironment structure and promote cell proliferation. Testicular cells were 3D bio-printed to show good cellular viability and maintain their original cellular properties in the body [97]. For human spermatogonial cells, the number of SSCs cultured on nanofiber porous scaffolds 3D-printed via electro-spinning was significantly increased compared with the control group, and this nanocomposite scaffold was proved to be able to maintain the self-renewal ability of human SSCs [114]. Another scaffold suitable for hybrid printing is the designed cell-loaded hydrogel, whose mechanical strength is also stable enough to support cell growth needs, after the cell-loaded hydrogel is 3D-printed [115], and hydrogels should have many desirable characteristics that are highly tunable; these were still to be considered during the printing process [116]. Therefore, in a recent new application in fish cell 3D printing, the gelatin-based gel is mixed with muscle stem cells for cell 3D printing culture, following the texture of fish muscle tissue [117]. This provides a good reference for the 3D printing of SSCs, using matrix glue to mix a variety of cells (such as Sertoli cells) to construct the microenvironment structure of SSC niches. At the same time, we should note that the special structure of SSCs’ niche microenvironment (as shown in Figure 1) is more complex than muscle tissue, which also puts higher requirements on the spatial model construction of 3D printing technology and the physical and chemical properties of the printing matrix.

## 4. Conclusions and Prospects

The demand for the long-term *in vitro* culture and research application of spermatogonial stem cells has prompted researchers to develop more convenient and efficient *in vitro* culture systems. DTM was designed to replace the traditional primary Sertoli cell feeder layer for the *in vitro* 3D culture of spermatogonial stem cells (Figure 4). Then, other simple biological substrates, such as agar, gelatin, and polysaccharides, were developed to replace DTM, as its preparation process is still cumbersome, and these developed culture systems have shown substantially the same results as the primary Sertoli cells, MEF cells, or DTM. In order to compensate for the lack of perfect reproduction of niche microenvironments in biological substrates, new 3D printing techniques have also been developed for the cultivation of SSCs *in vitro*. This culture system has also achieved good results. We think that with the development of cell engineering and biomaterials engineering technology, more and more efficient and convenient *in vitro* culture systems will be developed to simulate niche microenvironments for the long-term *in vitro* culture and transformation development of SSCs and other stem cells. To restore male fertility or maintain sperm vitality in the case of severe injuries (such as loss of SSCs in a teenager undergoing cancer treatment, severe testicular injury in a male due to accidental trauma, etc.), reproductive medicine is seeking more biotechnology alternatives, and one of the main methods adopted is the tissue reconstruction of bionic testis and flexure seminiferous tubules using tissue engineering principles and methods [113]. Through simulating the testis microenvironment and physiological conditions, the proliferation and differentiation of SSCs could be maintained *in vitro* culture, and male gametes could be produced for transplantation and recovery of reproductive function. Various biomaterials, from synthetic polymers to acellular substrates, have been applied, but each has its advantages and disadvantages in cell culture and tissue reconstruction.

Three-dimensional bioprinting niches that provide a suitable microenvironment for the cell is an essential issue to consider when designing scaffolds for three-dimensional cell culture. The ECM in the body offers an intricate nanoscale structure that supports cells and instructs them to control their properties [84]. Like in 2D culture, cells flatten and spread when combined with the micro-structure scaffold in 3D culture, and subtle nanoscale changes in the cell culture scaffold could also lead to different cell biology changes. Therefore, in addition to considering the size and structure of biological materials, factors such as the material, surface chemical properties, hardness, permeability, and mechanical force of natural materials during three-dimensional culture need to be carefully considered, which will have a significant impact on cell adhesion, proliferation, and biological behavior [118]. Dedicatedly designed hydrogels could be used as 3D cell culture scaffolds because they could mimic the properties of biological ECMs, and they could be bonded to define functional sites such as proteolytic sites and embedded growth factors, with their apparent chemical and physical properties, with adjustable mechanical properties and the required stiffness or porosity. At present, among the synthetic natural polymer hydrogels and unnatural polymer hydrogels, the unnatural polymer hydrogels are widely used and their structure is easy to adjust via synthesis or crosslinking, because of their relatively cheap, bioinert, and renewable characteristics, such as polyethylene glycol (PEG) and polylactic acid (PA) [114]. For example, PEG gels and their derivatives have been used in various cell cultures, including stem cell proliferation and differentiation, cell migration and invasion, and organoid angiogenesis [75]. However, non-natural polymers lack bonding parts in natural ECMs that require cross-linking peptides to improve function. Spermatogonial stem cells have been facing the problem of cell senescence during *in vitro* culture, and SSC cultures contain a heterogeneous group of cells as the SSCs also undergo partial differentiation *in vitro*. Long-term culture periods resulted in a loss in stem cell potential without an obvious change in the visual appearance of the culture, while DNA microarray analysis of *in vitro*-aged SSCs identified the differential expression of several genes important for SSC function, including B-cell CLL/lymphoma 6, member B (Bcl6b), Lim homeobox protein 1 (Lhx1), and thymus cell antigen 1, theta (Thy1) [119]. Because of the persistent cell cycle arrest caused by cell senescence, it is difficult for SSCs cultured *in vitro* to achieve long-term immobile culture [120]. Based on the characteristics of bioactive substances, the niche environment required for the construction of SSCs could be established [121], in which bioactive small molecules regulate SSCs to resist aging and promote proliferation and then immortality, such as rapamycin, resveratrol, and other inhibitors or activators of histone deacetylase (HDAC) family members [122,123,124].

Bioengineering using various biological materials could provide a variety of essential and innovative methods for tissue engineering and cell engineering-related research. To maintain the reproductive potential of humans and other species, the bioengineered *in vitro* culture system needs to highlight the function and role of the testicular microenvironment and spermatogenic cell niche environment [113]. Spermatogenesis is a complex process entirely dependent on the convoluted tubule microenvironment that supports cell participation in the construction. In the research of reproductive bioengineering, *in vitro* cell engineering has been trying and developing various biological materials that are conducive to the survival, proliferation, and differentiation of reproductive cells, including synthetic, natural, and extracellular matrix-derived materials and their derivatives. At present, these biomaterials have great potential for male reproductive bioengineering and medical applications such as tissue engineering, organoids, and new drug development, because they could be modified at the molecular level to develop new derivatives and more scalable resources. In order to solve a series of problems faced by *in vitro* induction and *in vivo* spontaneous spermatogenesis, some advanced engineering techniques are currently being used to manufacture more biocompatible biomaterials required for reproductive bioengineering, such as 3D scaffold bioprinting using hydrogels and acellular extracellular matrix. In the process of printing various types of cells on scaffolds, bio-printing technology has the advantage of freely controlling the shape of biological materials and the spatial arrangement of different types of cells, which are necessary factors lacking in previous cell engineering systems. In future studies, these biomaterials and new *in vitro* culture techniques could create and simulate more suitable testicular tissue and seminiferous microenvironments.
